# Evaluation of the biomarker candidate MFAP4 for non-invasive assessment of hepatic fibrosis in hepatitis C patients

**DOI:** 10.1186/s12967-016-0952-3

**Published:** 2016-07-04

**Authors:** Thilo Bracht, Christian Mölleken, Maike Ahrens, Gereon Poschmann, Anders Schlosser, Martin Eisenacher, Kai Stühler, Helmut E. Meyer, Wolff H. Schmiegel, Uffe Holmskov, Grith L. Sorensen, Barbara Sitek

**Affiliations:** Medizinisches Proteom-Center, Ruhr-Universität Bochum, 44801 Bochum, Germany; Department of Gastroenterology and Hepatology, Berufsgenossenschaftliches Universitätsklinikum Bergmannsheil, Bochum, Germany; Molecular Proteomics Laboratory (MPL), Biologisch-Medizinisches Forschungszentrum (BMFZ), Heinrich-Heine-Universität, Düsseldorf, Germany; Institute of Molecular Medicine, University of Southern Denmark, Odense, Denmark

**Keywords:** Hepatic fibrosis, Cirrhosis, Microfibrillar-associated protein 4, Serum biomarker, Hepatitis C, Direct acting antivirals

## Abstract

**Background:**

The human microfibrillar-associated protein 4 (MFAP4) is located to extracellular matrix fibers and plays a role in disease-related tissue remodeling. Previously, we identified MFAP4 as a serum biomarker candidate for hepatic fibrosis and cirrhosis in hepatitis C patients. The aim of the present study was to elucidate the potential of MFAP4 as biomarker for hepatic fibrosis with a focus on the differentiation of no to moderate (F0–F2) and severe fibrosis stages and cirrhosis (F3 and F4, Desmet-Scheuer scoring system).

**Methods:**

MFAP4 levels were measured using an AlphaLISA immunoassay in a retrospective study including *n* = 542 hepatitis C patients. We applied a univariate logistic regression model based on MFAP4 serum levels and furthermore derived a multivariate model including also age and gender. Youden-optimal cutoffs for binary classification were determined for both models without restrictions and considering a lower limit of 80 % sensitivity (correct classification of F3 and F4), respectively. To assess the generalization error, leave-one-out cross validation (LOOCV) was performed.

**Results:**

MFAP4 levels were shown to differ between no to moderate fibrosis stages F0–F2 and severe stages (F3 and F4) with high statistical significance (*t* test on log scale, *p* value <2.2·10^−16^). In the LOOCV, the univariate classification resulted in 85.8 % sensitivity and 54.9 % specificity while the multivariate model yielded 81.3 % sensitivity and 61.5 % specificity (restricted approaches).

**Conclusions:**

We confirmed the applicability of MFAP4 as a novel serum biomarker for assessment of hepatic fibrosis and identification of high-risk patients with severe fibrosis stages in hepatitis C. The combination of MFAP4 with existing tests might lead to a more accurate non-invasive diagnosis of hepatic fibrosis and allow a cost-effective disease management in the era of new direct acting antivirals.

**Electronic supplementary material:**

The online version of this article (doi:10.1186/s12967-016-0952-3) contains supplementary material, which is available to authorized users.

## Background

The hepatitis C virus (HCV) infection represents one of the main causes of chronic liver diseases worldwide [1]. Estimations of global HCV prevalence range from >185 million people with anti-HCV in 2005 (corresponding to 2.8 % of the population) [2] to 160 million people in 2013 [1]. Recently published data suggests that the global prevalence of viraemic HCV infections was 1.1 % in 2013 corresponding to 80 (64–103) million persons; with approximately 3.8 million viraemic infections in Western and Central Europe and 2.8 million infections in North America [3]. In recent years the treatment regimens for hepatitis C have changed dramatically. The new direct acting antivirals (DAAs) showed a higher efficiency and less side effects than previous interferon-based therapies thereby offering new treatment opportunities [4, 5]. Dependent on the HCV genotype, sustained viral response (SVR) rates of 40–80 % were reached with old treatment regimens whereas therapies with new DAAs promise SVR rates above 90 % [5]. On the other hand the extreme costs associated with the new drugs may overburden the healthcare systems which makes it necessary to first identify those patients which are in greatest need of therapy [6, 7].

The disease progression of hepatitis C infection is characterized by the development of hepatic fibrosis, but its course is highly variable. It ranges from minimal histological impairments to extensive fibrosis and cirrhosis with or without development of hepatocellular carcinoma (HCC) [8]. Currently, the European Association for the Study of the Liver (EASL) recommendations in 2015 on treatment of hepatitis C suggest prioritized treatment of patients with fibrosis stage F3 and F4 (METAVIR) while treatment is justified for stage F2. For stages F0 and F1 the timing of the therapy may be individualized [5]. Up to now, a liver biopsy is considered the gold standard for assessment of hepatic fibrosis although the procedure is invasive and carries a significant rate of complications, especially in patients with coagulation disorders (e.g. patients with advanced cirrhosis) [9]. Moreover, liver biopsy is prone to inter-observer-variability and sampling error as only a small fraction of the liver is analyzed [10]. To reduce the need of biopsy, many efforts were made in the last 15 years to develop non-invasive methods for assessment of hepatic fibrosis and imaging modalities to measure liver stiffness by transient elastography (TE) were developed (FibroScan, Echosens, Paris, France) [11]. Other tests such as FibroTest, also known as FibroSure [12] or the AST to platelet ratio index (APRI) [13] combine several serum parameters in scores to assess the stage of hepatic fibrosis. These diverse non-invasive tests have been extensively investigated in viral hepatitis [14] and showed an improved performance when used in combination [15]. However, several confounders must be taken in consideration for interpreting these tests and complicate a valid fibrosis assessment. One example is the commonly used FibroTest score, which is calculated using a blood test combining six serum markers with the age and gender of the patients. It is known that ribavirin therapy can induce hemolysis leading to decreased levels of haptoglobin, which is one of the six serum markers, therefore representing potential bias for the test. Furthermore, transient elastography is frequently not interpretable, especially in obese patients or those with ascites in decompensated cirrhosis [15]. This illustrates that improvement of existing tests is still necessary to ensure the valid diagnosis of hepatic fibrosis, which is essential for the therapeutic management of the hepatitis C infection.

To identify new biomarker candidates for hepatic fibrosis, we previously used a proteomics approach to analyze microdissected cirrhotic septae and liver parenchyma cells [16]. We detected an elevated abundance of extracellular human MFAP4 in the cirrhotic septae and recently confirmed the elevated expression of MFAP4 in hepatic fibrosis investigating tissue samples from a larger patient cohort [17]. The quantitative analysis of MFAP4 serum levels in hepatitis C patients by ELISA already demonstrated its applicability as a blood-based biomarker for non-invasive assessment of liver fibrosis. These results indicated a promising diagnostic accuracy for the prediction of non-diseased liver versus cirrhosis [16]. However, the statistical power was not sufficient to allow an accurate discrimination of no to moderate stages of hepatic fibrosis (F0–F2) from severe fibrosis and cirrhosis (F3 and F4). The aim of the present study was to assess the applicability of MFAP4 as a serum biomarker for hepatic fibrosis for the discrimination of fibrosis stages F0–F2 from fibrosis stages F3 and F4 investigating a large cohort of hepatitis C patients (*n* = 542). The performance of MFAP4 in this differentiation is of particular relevance as patients with severe fibrosis should immediately receive treatment with new direct acting antiviral drugs.

## Methods

### Clinical cohort

Serum samples collected by the German network of Excellence for Viral Hepatitis (HepNet, http://www.kompetenznetz-hepatitis.de) were used for the analysis of serum MFAP4 in patients with different stages of hepatic fibrosis. The study was approved by the local ethics committee and the procedure and the use of a part of the biopsy for this study were explained to the patients. All patients gave informed consent. The samples were collected at different sites using a standardized protocol reducing bias by inhomogeneous pre-analytical sample treatment. Patients with HCV RNA detectable by PCR who had undergone liver biopsy between 2001 and 2006 were included in the study. The resulting clinical cohort consisted of 555 samples in total and the collected patient information included covariates such as age, gender, HCV genotype or presence of co-morbid HCC. For the present analysis, we only considered patients with chronic HCV infection and available values for MFAP4 level, fibrosis, age and gender resulting in a sample size of *n* = 542. A descriptive analysis of the available relevant covariates can be found in Table 1 and Additional file 1.

**Table 1:** Patient cohorts characteristics, subdivided by fibrosis stage

Fibrosis stage^a^	F0	F1	F2	F3	F4	All stages
*Age* ^b^						
Mean age SD	43.75 ± 12.11	44.83 ± 12.64	51.24 ± 12.04	57.10 ± 10.49	57.33 ± 11.31	49.30 ± 13.08
*Gender* ^c^						
Women	52	85	66	36	28	267
Men	45	91	69	31	39	275
Number of patients	97	176	135	67	67	542
*HCV genotype* ^c^						
1	70	126	100	50	43	390
2	2	12	1	1	3	19
3	11	25	12	5	7	60
4	0	4	4	1	0	9
Other	0	1	0	0	0	1
NA	14	8	18	10	14	64

^a^Hepatic fibrosis was assessed by examination of liver biopsies by an experienced pathologist and staged according to Desmet-Scheuer scoring system

^b^For the age, the mean value per fibrosis stage is reported along with the corresponding standard deviation

^c^For gender and HCV genotype absolute frequencies are given

### Histologic staging

Biopsies were staged blindly according to the Desmet-Scheuer scoring [18] system by one pathologist with a specialization in liver pathology. Every biopsy specimen was staged on a scale of fibrosis F0 to F4: F0—no fibrosis; F1—enlarged, fibrotic portal tracts; F2—periportal fibrosis or portal–portal septa, but intact architecture; F3—fibrosis with architectural distortion, but no obvious cirrhosis; and F4—probable or definite cirrhosis.

### Sampling and laboratory measurements

MFAP4 was measured by AlphaLISA technique (PerkinElmer) as described in detail before [19]. Briefly, two monoclonal anti-MFAP4 antibodies (HG-HYB 7–14 and HG-HYB 7–18) that were generated using MFAP4-deficient mice [20] were used as acceptor and donor antibodies, respectively. Measurements were performed in 384 well format and all sera were tested in duplicates in a 1:100 dilution. When being measured in serum samples 1 U/ml MFAP4 corresponds to 38 ng/ml of MFAP4.

### Analysis of variance

We applied a log_2_ transformation to MFAP4 measurements in order to reduce the skewness and obtain a more symmetrical distribution. Based on the assessment of *q*–*q* plots normality could be assumed for the transformed data (data not shown). The association between fibrosis stage and observed MFAP4 levels was initially assessed by a two-group comparison of no to moderate fibrosis (F0–F2) vs. severe fibrosis and cirrhosis (F3, F4) by Student’s *t* test. A one-sided hypothesis was tested to reflect and eventually validate the assumption of higher values in severe fibrosis stages and cirrhosis. Equal variances were assumed after the log transformation. To achieve a comprehensive evaluation, we also analyzed the fibrosis stages individually, using ANOVA to test for differences in mean MFAP4 levels of the five fibrosis stages. Afterwards, Tukey’s‚ honest significant difference (HSD) method was used for pairwise comparisons between the individual stages in case of a significant result of the ANOVA. The chosen significance level in this work was 0.05.

### Logistic regression and diagnostic characteristics

Different classification models based on logistic regression (details below) were derived to distinguish patients with no to moderate fibrosis stages (F0–F2) from patients with severe fibrosis and cirrhosis (F3, F4). The discriminative power of the resulting models was assessed by means of the AUC value (area under the receiver operating characteristics (ROC) curve) and the corresponding 95 % confidence interval (CI) based on DeLong’s method. We considered the group of stages F3 and F4 as cases, and in the following analyses sensitivity is defined as the (true) probability of classifying samples from F3 and F4 correctly, while specificity is the (true) probability of assigning a sample of stages F0–F2 to the correct group.

The performance of the univariate model *y*_*uni*_ solely based on MFAP4 measurements was compared to the performance of a multivariate model *y*_*multi*_ that additionally accounts for age and gender of the patients. For these additional covariates, previous studies have shown an association with MFAP4 serum levels [20, 21]. After fitting the respective model, a cutoff was determined in order to define a binary classification rule for the assignment of samples to the group of either F0–F2 or F3 and F4. Generally, we used Youden’s criterion for the cutoff selection, which maximizes the sum of sensitivity and specificity. Confidence intervals for sensitivity and specificity are based on the assumption of underlying binomial distributions and represent the 95 % confidence level.

In the target clinical application, the future classifier will affect the identification of ‘high-risk’ patients with severe fibrosis and cirrhosis (F3, F4). Thus, a lower limit for sensitivity (correct classification of F3 and F4) was considered, which translated into the analysis of a partial AUC (pAUC): The optimized cutoff was determined in a predefined range of sensitivity (0.80–1.00), using Youden’s criterion as for the traditional approach. We list sensitivity and specificity for Youden-optimal cutoffs resulting from the unrestricted analysis as well as for a minimum sensitivity value of 0.8. Note that these diagnostic values are prone to overoptimism, owing to the optimization process. In order to assess the generalization error, leave-one-out cross validation (LOOCV) was performed for each of the modelling approaches (restricted/unrestricted × univariate/multivariate). The values of sensitivity and specificity obtained from the LOOCV analysis are valid estimates for the true characteristics that the classifier would achieve on future independent cohorts. In the leave-one-out cross validation, the approach of interest (here fit of logistic model and choice of classification cutoff) is applied to the data set which was reduced by a single sample. The resulting model is then used to predict the class assignment (high or low fibrosis stage) of the left-out sample. With *n* being the total number of samples, this process is carried out *n* times, leaving out a different sample each time. The sensitivity and specificity of the LOOCV procedure are given by the proportion of correctly classified samples from high and low fibrosis stages, respectively.

## Results

### Analysis of variance

**Fig. 1:**
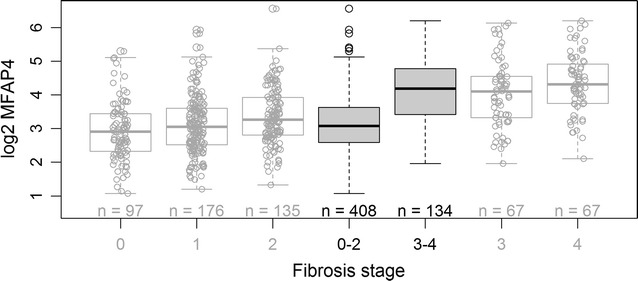
Two group comparison for the differentiation of no to moderate (F0–F2) and severe (F3, F4) fibrosis stages. *Upper* and *lower* *bounds of* *boxes* represent the first and third quartile per group, *whiskers* extend to the most extreme data point which is no more than 1.5 times the interquartile range from the box. *Circles* represent individual data points. *Solid grey* *boxes* represent the summarized two experimental groups tested for statistical significance by Student’s *t* test *(p* value < 2.2·10^−16^), while the *white boxes* represent the individual fibrosis stages F0–F4 (Desmet-Scheuer score). For reasons of clarity, the box plots for the combined groups (0–2 vs. 3–4) do not display the individual data points as the box plots for the individual groups

According to current EASL recommendations we focused on the differentiation of no to moderate fibrosis stages (F0–F2) and severe fibrosis and cirrhosis (F3, F4) by means of (log_2_) MFAP4 measurements (Fig. 1). We observed high statistical significance for this comparison (*t* test: *p* value <2.2*10^−16^ assuming equal variances) with mean values 3.15 (log_2_ U/ml) for stages F0–F2 and 4.19 (log_2_ U/ml) for stages F3 and F4. In addition, we tested for differences in means of the individual fibrosis stages by ANOVA and Tukey’s HSD post hoc test. We observed statistically significant differences except for comparisons of adjacent stages within the classes low and high, i.e. stages F0 vs. F1, F1 vs. F2 and F3 vs. F4. All results are summarized in Table 2.

**Table 2:** Results of the pairwise comparisons of individual hepatic fibrosis stages with respect to log_2_ MFAP4 values after significant ANOVA result

Comparison^a^	Difference^b^	Lower bound^c^	Upper bound^c^	*p* value^d^
F1–F0	0.1924	−0.1114	0.4962	0.4144
F2–F0	0.4386	0.1188	0.7584	0.0018
F3–F0	1.1168	0.7351	1.4984	3.43E−10
F4–F0	1.4277	1.0461	1.8092	3.43E−10
F2–F1	0.2462	−0.0287	0.5211	0.1036
F3–F1	0.9244	0.5796	1.2693	3.51E−10
F4–F1	1.2353	0.8903	1.5802	3.43E−10
F3–F2	0.6782	0.3191	1.0373	3.280E−6
F4–F2	0.9891	0.6300	1.3482	3.45E−10
F4–F3	0.3109	−0.1042	0.7260	0.2438

^a^Pairwise comparison between individual fibrosis stages

^b^Estimated difference between true mean values of individual groups (log_2_ U/ml)

^c^Upper respectively lower bound of the 95 % confidence interval of difference (log_2_ U/ml)

^d^Adjusted *p* value according to Tukey’s‚ honest significant difference’ post hoc test

### Diagnostic characteristics

**Fig. 2:**
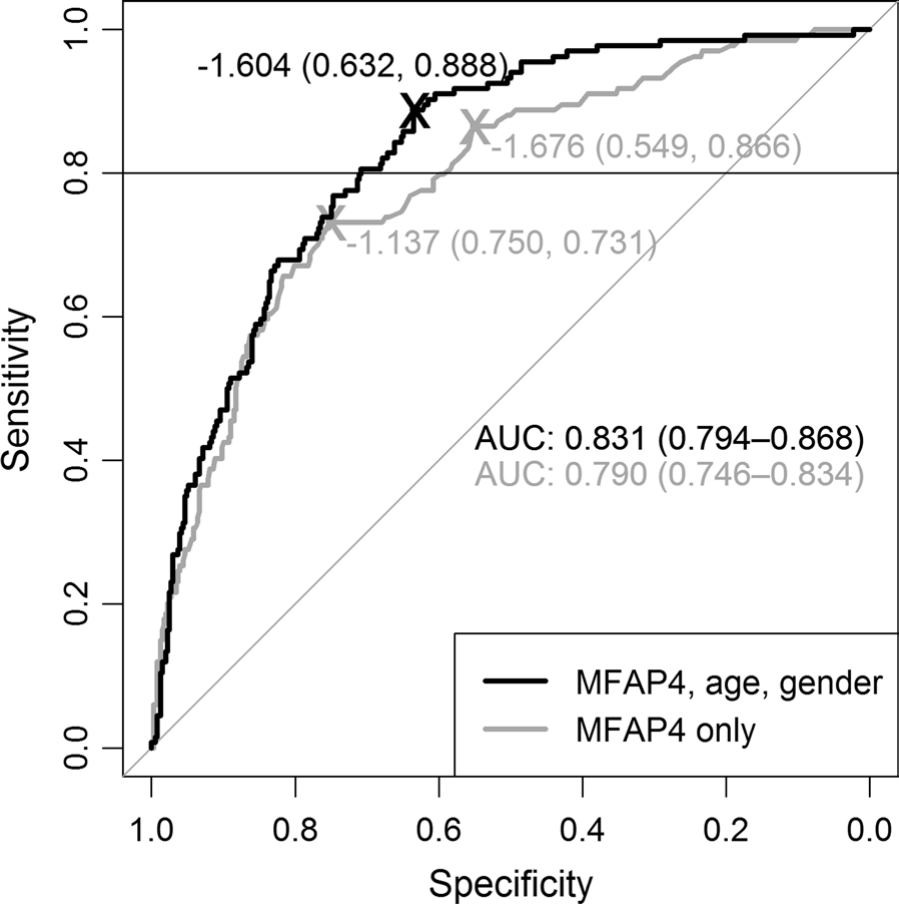
ROC curves based on univariate logistic regression model considering solely MFAP4 serum concentrations (*grey line*) and multivariate model considering also age and gender (*black line*), respectively. MFAP4 serum concentrations were measured in patients with different stages of hepatic fibrosis (F0–F4 according to Desmet-Scheuer-scoring system). The ROC curves represent the differentiation of mild to moderate (F0–F2) and severe fibrosis and cirrhosis stages (F3, F4). AUC values represent the area under the respective curves, values in brackets represent the 95 % confidence intervals. The given cutoffs are Youden-optimized with no restriction and within a sensitivity range of 0.8–1 indicated by the *horizontal line* on *top of the graph*, respectively. As the ROC curves represent the complete data set, corresponding diagnostic characteristics indicated at the optimal cutoffs are prone to overoptimism. Please refer to text for LOOCV results

To estimate the diagnostic potential of MFAP4 we performed ROC curve analysis and determined the optimal cutoffs for the discrimination of fibrosis stages F0–F2 and F3 and F4 using two different logistic regression models. First, we report the diagnostic characteristics estimated on the complete data set: The application of the univariate regression model *y*_uni_, with

$$y_{\rm uni} = - 5.50 + 1.20 \log_{2} MFAP4$$

yielded 73.1 % sensitivity and 75.0 % specificity (AUC 0.790, Fig. 2). The optimization considering a minimum of 80 % sensitivity resulted in a sensitivity of 86.6 % and a decreased specificity of 54.9 %. For the univariate models, these diagnostic values barely decreased in the cross validation: The LOOCV resulted in 71.6 % sensitivity and 75 % specificity for the unrestricted model and 85.8 % sensitivity and 54.9 % specificity when a minimum sensitivity of 80 % was considered. All estimated values are summarized in Table 3 along with the corresponding CIs.

**Table 3:** Sensitivity and specificity of the univariate and multivariate classification, respectively

	Multivariate model^a^		Univariate model	
	Unrestricted	Lower limit of 80 % sensitivity^b,c^	Unrestricted	Lower limit of 80 % sensitivity^b,c^
Sensitivity^c^ estimate [95 % CI]	88.8 % [82.2 %; 93.6 %]	88.8 % [82.2 %; 93.6 %]	73.1 % [64.8 %; 80.4 %]	86.6 % [79.6 %; 91.8 %]
Specificity^d^ estimate [95 % CI]	63.2 % [58.4 %; 67.9 %]	63.2 % [58.4 %; 67.9 %]	75.0 % [70.5 %; 79.1 %]	54.9 % [49.9 %; 59.8 %]
*Leave-one-out cross validation* ^e^				
Sensitivity^c^ estimate [95 % CI]	80.6 % [72.9 %; 86.9 %]	81.3 % [73.7 %; 87.5 %]	71.6 % [63.2 %; 79.1 %]	85.8 % [78.7 %; 91.2 %]
Specificity^d^ estimate [95 % CI]	61.5 % [56.6 %; 66.3 %]	61.5 % [56.6 %; 66.3 %]	75.0 % [70.5 %; 79.1 %]	54.9 % [49.9 %; 59.8 %]

^a^A multivariate logistic regression model considering age and gender besides MFAP4 serum levels was derived and the respective Youden optimal cutoffs were determined

^b^Cutoff optimization was restricted to a minimum sensitivity of 80 % accounting for the importance of the identification of high fibrosis stages

^c^Sensitivity was defined as probability of classifying stages F3 and F4 correctly

^d^Specificity was defined as probability of classifying stages F0 to F2 correctly

^e^As sensitivity and specificity values are prone to overoptimism in the analysis of the complete data set leave-one-out cross validation was performed to obtain unbiased estimates

The derived multivariate logistic regression model was calculated as follows:

$$y_{\rm{multi}} = - 7.31 + 1.02 \log_{2} {\rm {MFAP4}} - 0.51 I_{\rm {female}} + 0.05\, \rm{age},$$

where *I*_female_ is 1 if the sample to be classified is from a female patient and 0 otherwise.

The application of the multivariate model resulted in 88.8 % sensitivity and 63.2 % specificity (AUC 0.831) both when estimated without restrictions and when a lower limit of 80 % sensitivity was considered (Fig. 2). For the multivariate model the LOOCV resulted in 80.6 % sensitivity when the analysis was unrestricted and 81.3 % sensitivity when the lower limit of 80 % sensitivity was applied. For both approaches the specificity was 61.5 %. A summary of all estimated values is given in Table 3. In summary, the AUC increases with the inclusion of the previously reported confounders age and gender into the logistic model. Notably, the additional area is related to an increased sensitivity in the range of lower to medium specificity. This is especially beneficial in the given clinical setting, where we focus on high sensitivity to identify patients in need of immediate treatment. Comparing the diagnostic values for sensitivity and specificity obtained from the LOOCV analysis, the restricted multivariate classification model also yields the higher Youden index than the univariate.

## Discussion

According to the current EASL recommendations, the stage of hepatic fibrosis is a key factor to identify patients who should receive immediate treatment with new DAAs [5]. Hence, the importance of identification of patients with advanced fibrosis stages is obvious. Several non-invasive tests have been developed and each has specific advantages and disadvantages [22]. Measuring liver stiffness by TE is widely used and performs well in identification of cirrhosis. However, TE has been shown to be frequently not interpretable [15]. Furthermore, TE is generally limited by user experience [23], and requires a specific device. Serum tests are either based on indirect or direct markers. Indirect markers are parameters which are assessed in routine diagnostics and are combined into specific scores, some of which are protected by their commercial providers and therefore only limited available (e.g. FibroTest, Hepascore [24], Fibrometer [25]). Indirect markers are usually not disease specific and might produce false positive results due to comorbidities, e.g. in acute hepatitis if aspartate aminotransferase levels are included in the scoring algorithms (e.g. APRI, Fibrometer). Direct markers, such as TIMP metallopeptidase inhibitor 1 (TIMP1), hyaluronate [26] or metalloproteinases (MMP1, MMP3; e.g. FibroSpectII [27], ELF score [28]) reflect the processes of ECM remodeling, which take place in the liver. Therefore, direct markers seem to consolidate the rationale of a given test, when they are combined with indirect markers. Although the combination of several blood tests has been demonstrated to enhance the diagnostic accuracy [15, 29, 30], an ideal test does not yet exist and further improvement of the diagnostic tools is still necessary. In the present study, we examined the potential of MFAP4 to serve as a new serum biomarker for the assessment of hepatic fibrosis. In line with current treatment guidelines we emphasized the differentiation of patients with no to mild fibrosis (F0–F2) from severe fibrosis and cirrhosis (F3, F4).

MFAP4 is located in the extracellular matrix (ECM) and expressed in association with ECM fibers including elastic fibers in the entire body [19]. It also has been suggested to be involved in elastic fiber formation [31, 32]. Although only little is known about its molecular function, the relevance of MFAP4 is well-established for diseases associated with remodeling of the ECM such as vascular stenosis [33] and liver fibrosis [16, 17]. We demonstrated that MFAP4 plasma levels correlate with TE measurements and are significantly increased in patients with chronic HCV infection [21]. Moreover, MFAP4 has been shown to be involved in respiratory diseases [34–37]. However, recent data suggests that MFAP4 is not elevated in sera of patients with idiopathic pulmonary fibrosis (IPF) [38]. To assess MFAP4 serum levels for diagnostic purposes we established and further developed an immunoassay and examined the characteristics of MFAP4 in this context. MFAP4 was shown to be robust against variations in sample handling and storage conditions including repeated freeze and thaw cycles indicating ideal properties for usage in a clinical setting [20]. Several confounders that influence MFAP4 serum levels, such as age, sex, waist to hip ratio and smoking habits were already determined [20]. Several known confounders had not been acquired for the clinical cohort we used in the present retrospective study except for age and gender. For these, our results confirmed significant influence on (log_2_) MFAP4 serum levels in a multivariate logistic regression model. Furthermore, a lack of knowledge about patient’s comorbidities known to be capable of influencing MFAP4 serum levels has the potential to bias our results. Previously, we identified various cardiovascular conditions as well as COPD to be correlated with elevated MFAP4 serum levels [19, 35]. Yet, in a recent study only congestive heart failure was found to be associated with significantly increased MFAP4 plasma levels [21]. In addition, the cohort used in this study was not homogeneous regarding previous treatment. Parts of the cohort received IFN-based therapy with or without combination with ribavirin. However, the data were incomplete regarding therapeutic measures. In future prospective studies, the complete set of known confounders needs to be acquired and considered in a classification model for a thorough analysis. In consequence, we expect an improved model with increased diagnostic accuracy.

The established non-invasive tests for assessment of liver fibrosis have been extensively evaluated in the past [39, 40] and prospective studies allowing assessment of more relevant parameters were performed. Most established tests show a relatively good performance in prediction of cirrhosis (F4) but perform less well in diagnosis of severe fibrosis (F3). However, the accurate diagnosis of severe fibrosis and cirrhosis is the key requirement for non-invasive tests right now [5]. For prediction of fibrosis stages F3 and F4 (METAVIR) FibroScan showed an AUC of 0.75 (sensitivity 89.7 %, specificity 32.2 %) in a cohort of HCV patients [41]. Recently, the most common blood tests were evaluated for diagnosis of severe fibrosis in a prospective study showing AUCs of 0.82 for Fibrometer and Hepascore, respectively. APRI showed an AUC of 0.76 and the ELF score yielded an AUC of 0.78 [15]. Notably, we achieved comparable values using both either the univariate model (85.8 % sensitivity and 54.9 % specificity, AUC 0.79) or the multivariate model (81.3 % sensitivity and 61.5 % specificity, AUC 0.83) for diagnosis of fibrosis stages F3 and F4 in a more heterogeneous cohort. As these estimates are obtained from the LOOCV these estimates are basically unbiased. However, none of the established non-invasive tests could be applied to our same samples set, hence only an indirect comparison of the tests was possible and ultimate conclusions are still pending.

As patients suffering from severe fibrosis and cirrhosis (F3, F4) need to receive immediate treatment, which however is unfortunately often limited by the high cost associated with the DAAs, we considered the correct diagnosis of these patients to be of highest relevance in a clinical setting. In the class of lower fibrosis stages, one should further distinguish between misclassification of stages F0–F1 and stage F2, as immediate treatment is also justified for the latter one, but less important than for F3 and F4. A false diagnosis of no to moderate (F0–F2) might lead to immediate treatment, which is generally beneficial for the patients but involves high expenses. Therefore, we used an alternative approach, which focuses on the reliable diagnosis of patients with fibrosis stages F3 and F4 by calculating the optimized cutoffs for a minimum sensitivity (correct diagnosis stages F3 and F4) of 80 %. To our opinion, this is a reasonable choice for a minimum sensitivity and still yields acceptable values for specificity. For this lower bound the optimal classifier resulted in 85.8 % sensitivity and 54.9 % specificity for the univariate model. 81.3 % sensitivity and 61.5 % specificity were reached applying the multivariate model (Table 3, LOO cross validated values). These values represent the correct identification of 85.8 and 81.3 % of patients in the greatest need of immediate therapy. While for the multivariate model the sensitivity is slightly decreased compared to the univariate one, the specificity increased (61.5 % compared to 54.9 %). Here, decreased specificity means that patients with lower fibrosis stages (F0–F2) are misclassified, thus, they are suggested for DAA treatment. As immediate treatment is justified for F2 patients, their misclassification increases overall treatment expenses but still is in line with EASL recommendations. We therefore assessed the misclassification rates for the lower fibrosis stages separately and F2 turned out to have the highest misclassification rate of about 50 %, which qualifies the rather low values for specificity reported above.

## Conclusions

We were able to demonstrate the applicability of MFAP4 as a serum biomarker for hepatic fibrosis in a large cohort of hepatitis C patients. Both, a univariate logistic regression model considering solely MFAP4 serum levels as well as a multivariate model taking into account also age and gender were applied. Albeit a great heterogeneity of the analyzed cohort must be assumed, both models resulted in sensitivity and specificity estimates comparable with existing tests. We showed that our models facilitate the reliable identification of patients with severe fibrosis stages (F3 and F4) reflecting the need of prioritized treatment of these patients according to the current EASL recommendations. In consequence, MFAP4 may prospectively help improving the cost-effective management of the hepatitis C-infection and the disease specific complications, considering the necessity of immediate therapy of high-risk patients.

## Additional file

10.1186/s12967-016-0952-3 Clinical cohort characteristics and MFAP4 serum levels.

## Abbreviations

MFAP4: microfibrillar-associated protein 4
ECM: extracellular matrix
HCV: hepatitis C virus
SVR: sustained viral response
DAA: direct acting antivirals
EASL: European Association for the Study of the Liver
HCC: hepatocellular carcinoma
TE: transient elastography
AST: aspartate aminotransferase
APRI: AST to platelet ratio index
ELISA: enzyme linked immunosorbent assay
LOOCV: leave-one-out cross validation
GGT: gamma-glutamyl transpeptidase
ALT: alanine transaminase
TIMP: tissue inhibitor of metalloproteases
TIMP1: TIMP metallopeptidase inhibitor 1
MMP: matrix metalloproteinase
AUC: area under the curve
ROC: receiver operating characteristics
CI: confidence interval
ELF: enhanced liver fibrosis
COPD: chronic obstructive lung disease
IPF: idiopathic pulmonary fibrosis
IFN: interferon
